# Influence of sildenafil on blood oxygen saturation of the obstructed bladder

**DOI:** 10.1186/1471-2490-14-44

**Published:** 2014-05-29

**Authors:** Jeroen R Scheepe, Arjen Amelink, Katja P Wolffenbuttel, Dirk J Kok

**Affiliations:** 1 Department of Urology and Pediatric Urology, Erasmus Medical Center Rotterdam, Sophia Children’s Hospital, Rotterdam, The Netherlands; 2 Department of Radiation Oncology, Center for Optical Diagnostics and Therapy, Erasmus Medical Center, Rotterdam, The Netherlands

**Keywords:** Bladder dysfunction, Bladder outlet obstruction, Guinea pig, Hypoxia, PDE5 inhibitor

## Abstract

**Background:**

Blood oxygen saturation (BOS) is decreased in a low-compliant, overactive obstructed bladder. The objective of this study is to determine the effect of Sildenafil (SC) on bladder function and BOS) in an in vivo animal model of bladder outlet obstruction.

**Methods:**

Thirty-two guinea pigs; sham operated (n = 8), sham operated + SC (n = 8), urethrally obstructed (n = 8) and urethrally obstructed + SC (n = 8) were studied during an 8 week period. BOS of the bladder wall was measured by differential path-length spectroscopy (DPS) before obstruction, at day 0, and at week 8. The bladder function was evaluated by urodynamic studies every week.

**Results:**

Before surgery and after sham operation all study parameters were comparable. After sham operation, bladder function and BOS did not change. In the obstructed group the urodynamic parameters were deteriorated and BOS was decreased. In the group obstruction + SC, bladder compliance remained normal and overactivity occurred only sporadic. BOS remained unchanged compared to the sham group and was significantly higher compared to the obstruction group.

**Conclusions:**

In an obstructed bladder the loss of bladder function is accompanied by a significant decrease in BOS. Treatment of obstructed bladders with SC yields a situation of high saturation, high bladder compliance and almost no overactivity. Maintaining the microcirculation of the bladder wall might result in better bladder performance without significant loss of bladder function. Measurement of BOS and interventions focussing on tissue microcirculation may have a place in the evaluation / treatment of various bladder dysfunctions.

## Background

There is growing evidence that ischemia of the bladder wall contributes to the initiation of bladder dysfunction. Several studies have shown effects in obstructed bladder that can be interpreted as long term results of hypoxia [1]. Furthermore, there is an increasing interest in the nitric oxide (NO) pathway as a potential pharmacological target to treat lower urinary tract symptoms.

Phosphodiesterase 5 (PDE5) is involved in the NO pathway and it has been immunolocalized both in the detrusor muscle cells and in the vascular endothelium [2]. PDE5 inhibitors were found to improve several functional aspects of bladder dysfunction in human and animal studies [2-10]. The mechanisms behind this have not been fully elucidated yet, but it can be assumed that PDE5 inhibitors influence detrusor muscle cell action directly but also indirectly through enhancement of tissue microcirculation of the bladder tissue [11]. In a previous study we have shown in a Guinea pig model of bladder outlet obstruction (BOO) that the oxygen saturation is significantly lower in the obstructed bladder compared to sham operated bladder, both during the voiding and filling phase [12].

The objective of this study is to investigate the effect of the PDE5 inhibitor Sildenafil citrate (SC), which enhances tissue microcirculation, on bladder function and blood oxygen saturation (BOS) *in vivo* in an animal model of bladder outlet obstruction.

## Methods

### Animals and study design

Animal experiments were approved by the Erasmus Medical Center animal ethics committee. Furthermore, the experiments were conducted according to the ARRIVE guidelines.

Thirty-two immature male albino Guinea pigs (Hartley strain) weighing approximately 250 g were used. Sixteen animals were urethrally obstructed and 8 of them received daily s.c. injections with sildenafil citrate (SC) (10 mg/kg b.w./day). The other 8 animals received saline only. Another group of 16 animals were sham operated and also divided into two groups: plus SC (n = 8) and plus saline (n = 8). All 32 animals were followed for 8 weeks. Urodynamic investigations were performed before surgery and at weeks 2,3,4,5,6,7 and 8. DPS measurements were performed at day 0 before surgery (n = 32) and 8 weeks after sham operation (n = 16) or obstruction (n = 16).

### Experimental model, surgical procedures and DPS measurements

The Guinea pig model for partial bladder outlet obstruction (BOO) as described by Kok and Wolffenbuttel et al. [13,14] was used.

Obstruction and sham operation were done using ketamine/xylazine anesthesia. The peritoneal cavity was accessed via a lower vertical midline abdominal incision. A silver jeweler jump ring with an internal diameter of 2.2 mm was placed around the bladder neck above the prostate and left there (obstructed group) or removed (sham operated group). A glass fiber probe was then placed directly on the body of the bladder for BOS measurements. At the day of sacrifice a similar midline incision was made to allow probe access to measure BOS of the bladder wall, as described below, during multiple filling/voiding cycles. Intravesical pressure was measured simultaneously. The flow rate was not measured during DPS measurements but each DPS measurement sequence was preceded by a complete urodynamic investigation, including flow rate measurement. After the final DPS measurement the animal was sacrificed and the bladder was removed en bloc in order to determine the bladder weight.

### Urodynamics

Urodynamic investigations were performed at week 0 (before the obstruction/sham operation and first DPS measurement sequence), week 2, 3, 4, 5, 6, 7 and at week 8 (before the second DPS measurement sequence). For each measurement the animals were anesthetized using ketamine (43 mg/kg i.m.) and xylazine (0.9 mg/kg i.m.). Through a 24-gauge suprapubic catheter bladder pressure was measured and the bladder was filled continuously with sterile saline at a rate of 0.23 ml per minute. Flow rate was measured with an ultrasound transducer (T106 small animal Flow meter, Transonic Systems, Ithaca, NY) around the penis.

From the urodynamic data we calculated:

– 1) Number of overactive contractions (NOC): Number of overactive contractions (>10 cm H_2_O) that occur during 1 filling cycle. The average NOC of all cycles during 1 urodynamic investigation is reported.

– 2) Maximum voiding pressure (P_max_ in cm H_2_O): Average P_max_ of all voids during 1 urodynamic investigation is reported.

– 3) Contractility (W_max_ in W/m^2^): Relation between pressure and flow during a voiding according to Griffiths et al. [15]. The average W_max_ for all voidings during 1 urodynamic investigation is reported.

– 4) The maximal flow rate (Q_max_ in ml/sec): Highest absolute value of the flow during voiding.

– 5) Bladder compliance (ml/cm H2O) was defined as the relationship between change in bladder volume and the change in bladder pressure in the filling phase. Care was taken that pressure values obtained during these periods were not influenced by a nearby voiding or overactive contraction.

### Differential path-length spectroscopy (DPS)

Blood oxygenation of the bladder wall was measured *in vivo* by differential path-length spectroscopy (DPS) using glass-fibers at the 2 time points where the bladder was accessible. During concomitant bladder pressure measurement the probe was placed in gentle contact with the serosal surface of the anterior bladder wall. To avoid artefacts caused by pressing the probe too hard to the bladder wall the probe was regularly repositioned. The experimental setup used for DPS measurements and the DPS data analysis routine was previously described in detail by Amelink et al. [16,17]. Complete sessions consisted of a few hundred to more then a thousand single DPS measurements. During voiding, up to 10 DPS measurements and during filling, up to hundreds of DPS measurements could be performed. The average saturation was calculated when at least 5 measurement points were available.

### Statistics

We tested the significance of changes at the 2 time points in the obstructed and sham operated groups with the paired Student t test. Differences between the obstructed and sham operated groups were tested with the unpaired Student t test.

## Results

### Loss of animals

Two animals from the obstruction group with saline developed bladder stones and had to be removed from the study.

In all SC treated animals the urodynamic investigation at week 5 was not performed due to organizational problems.

### Urodynamic data and BOS before operation

**Figure 1:**
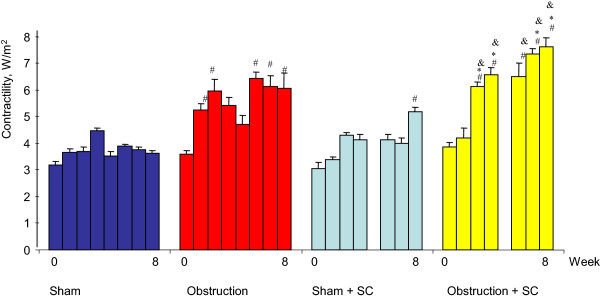
**Bladder contractility during the 8 week period.** *significance of difference vs sham, p < 0.05, & significance of difference vs obstruction, *significance of difference versus sham + sildenafil.

**Figure 2:**
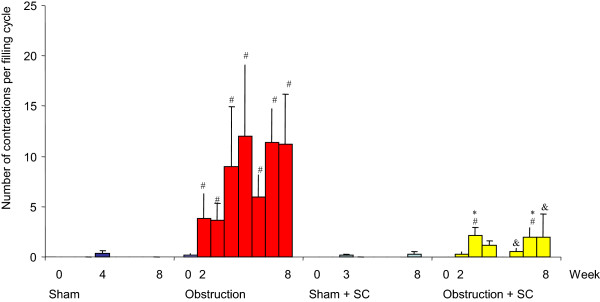
**Bladder over**-**activity during the 8 week period.** #significance of difference vs sham, p < 0.05, & significance of difference vs obstruction, *significance of difference versus sham + sildenafil.

**Figure 3:**
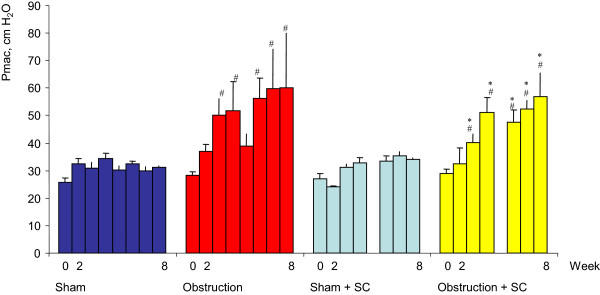
**Maximum voiding pressure during the 8 week period.** #significance of difference vs sham, p < 0.05, & significance of difference vs obstruction, *significance of difference versus sham + sildenafil.

**Figure 4:**
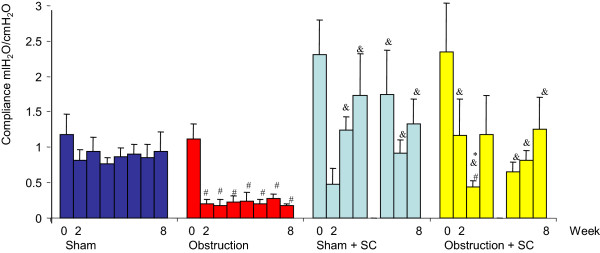
**Bladder compliance during the 8 week period.** #significance of difference vs sham, p < 0.05, & significance of difference vs obstruction, *significance of difference versus sham + sildenafil.

Before obstruction or sham operation there were no statistically significant differences between the 4 groups concerning contractility (±3 W/m^2^), overactivity (no unstable contractions) and maximum voiding pressure (<30 cm H_2_O) (Figures 1, 2 and 3). The average value for bladder compliance was higher in the two SC treated groups but this difference was not significant (Figure 4). Flow rate was comparable in all 4 groups (data not shown).

**Figure 5:**
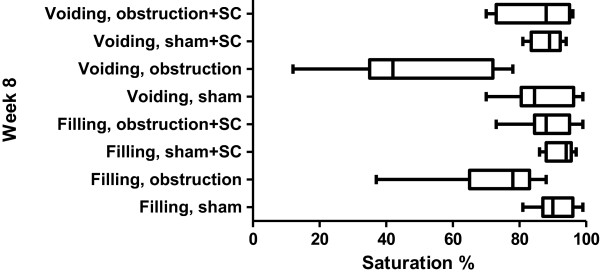
**The % saturation during filling and voiding before sham/obstructive surgery.** The bars represent the lowest, highest and average value.

The BOS of the bladder during filling averaged between 92% and 95% in the four groups (Figure 5). The complete range was moved slightly to higher values in the two SC treated groups but all differences between the groups were not statistically significant. During voiding the average BOS ranged from 84% to 94% in the 4 groups. The individual BOS values showed more variation, ranging from 62% to 98% (Figure 5).

### Urodynamic data and BOS after sham operation

**Figure 6:**
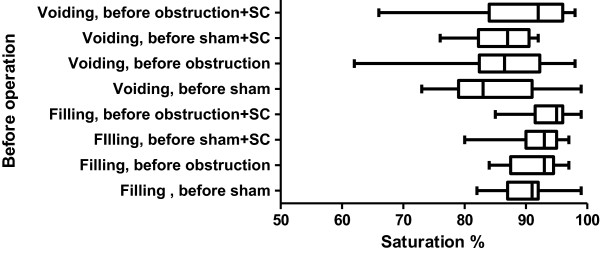
**The % saturation during filling and voiding 8 weeks after sham/obstructive surgery.** The bars represent the lowest, highest and average value.

In both the sham + saline and sham + SC group the maximum voiding pressure, compliance, contractility and overactivity did not change during the 8 week follow-up. A few unstable contractions were found in some animals from both groups at weeks 4 and 8. No differences were found between both groups except for a statistically significant increase in contractility at week 8 in the sham + SC group (Figure 1). At week 8 BOS was comparable between both groups and unchanged from the values at day 0 (Figure 6).

### Urodynamic data and BOS after obstruction

In the *obstruction* + *saline group* bladder compliance was significantly lower compared to both day 0 and to the corresponding value in the sham + saline group from week 2 up until week 8. Maximum voiding pressure increased compared to day 0 and to the sham + saline group, reaching significance at weeks 3,4,6,7 and 8. Bladder overactivity was present from week 2 onwards. Bladder contractility was higher compared to day 0 and to the sham + saline group, reaching significance at weeks 2,3,6,7 and 8. The average value for BOS decreased significantly both in the filling and voiding phase. The lower limit of individual voids was 12% (Figure 6).

In the *obstruction* + *SC group* compliance remained comparable to the values at day 0 and to the values in both sham groups. Compared to the obstruction + saline group the compliance was significantly higher at weeks 2,3,6,7 and 8. The maximum voiding pressure was similar to the obstruction + saline group and significantly higher compared to day 0 and to the sham groups. Contractility was increased significantly compared to day 0 and to both sham groups and even compared to the obstruction + saline group (weeks 3,4,6,7 and 8). Overactivity was found throughout the 8 week follow-up but was significantly lower as compared to the obstruction + saline group.BOS did not decrease in the obstruction + SC group after 8 weeks of obstruction. It was significantly higher compared to the obstruction + saline group. The lowest individual value was 71% (Figure 6).

### Bladder weight

The average bladder weights of the sham + saline group and obstruction + saline group was 0.75 ± 0.07. and 1.64 ± 0.36 grams, respectively (p < 0.04). Treatment of the obstructed animals with SC resulted in a significant lesser increase of bladder weight (0.91 ± 0.1 gr.). The bladder weight of the sham + SC group was lower (0.55 ± 0.03) compared to the sham + saline group.

## Discussion

During bladder filling the saturation in the normal Guinea pig bladder is around 90%. At the end of bladder filling and during voiding there is a marked decrease in saturation. Shortly after voiding the saturation returns to its high pre-voiding value.

The obstructed bladder is characterized by a significantly lower saturation both during voiding and filling. This decrease in saturation is more pronounced when detrusor overactivity occurs during the filling phase [12].

In contrast, in Guinea pigs with BOO treated with SC the bladder saturation both during filling and voiding remains as high as in the sham operated animals. This maintenance of a high saturation level is accompanied by normal bladder compliance and less overactivity. Maintaining bladder compliance and damping detrusor overactivity might be explained by direct smooth muscle cell action of the PDE5 inhibitor SC. However, the maximum voiding pressure and the contractility of SC-treated obstructed bladder increase at least as much as in the saline treated obstructed bladder. This phenomenon can not be explained by a direct muscle relaxing effect of SC alone. Possibly the maintenance of an almost normal bladder function in animals with BOO treated with SC is an effect of enhanced bladder microcirculation rather then a direct effect on muscle cells. Obstructed bladders treated with SC have a higher saturation level.. Muscle cells acting aerobically can produce force more efficiently than the same amount of muscle cells acting under anaerobic circumstances. As a consequence, the increased contractility and voiding pressure that are needed to overcome the obstruction need less muscle mass and the bladder wall increases less in size resulting in less muscle hypertrophy. This may partly explain the better compliance and the lower bladder weight that was observed in the SC treated obstructed bladder. Low saturation may excite nerve endings in the bladder wall, leading to overactivity and higher muscle tone. Maintenance of a high saturation could prevent this nerve action and may result in better bladder compliance. In support of this is our finding that SC treated obstructed bladder shows less overactivity.

Similar effects of PDE5 inhibitors on bladder function have been found with *in vitro* muscle strip tests in a rat model of BOO [18,19]. The carbachol induced contractile force of bladder strips tested *in vitro* is reduced by BOO. Vardenafil treatment during the obstructive period diminishes this in vitro loss of carbachol induced contractility [18]. Muscle strips from sham operated rats that received vardenafil showed an increased contractility as compared to normal rat bladder strips [19]. In our experiments, we noticed a trend for increased contractility of the whole bladder in the sildenafil treated sham operated animals but this was only significant at week 8. This might be explained by a slight increase of bladder saturation during detrusor contraction. Furthermore, in the sildenafil treated sham operated animals the bladder weight was lower compared to sham + saline group. Thus, possibly the unobstructed bladder also benefits from optimal saturation.

In our experimental setup we did not determine a tissue marker for hypoxia such as glycogen or hypoxia-inducible factor 1 (HIF-1). In a previous study we demonstrated that the presence of glycogen deposits in the bladder wall correlates well with loss of bladder function in both Guinea pigs and humans [20,21]. Furthermore, we demonstrated in another study [12] that the glycogen content of the bladder wall was increased in obstructed Guinea pigs in comparison with sham operated animals. The same study revealed a good correlation between DPS measurements and glycogen deposits in the bladder wall. Therefore, we did not expect any further relevant information from tissue markers in this experimental setup and determination of glycogen deposits were not done on a regular basis.

There is evidence that oxidative stress is a key feature in the initiation and progression of voiding dysfunction [1]. Although the data are preliminary, they correlate with a mechanism where bladder dysfunction in BOO is initiated by bladder pressure related ischemia and reperfusion injury. With the present model it is not obvious if the effect is just a matter of better flow due to vessels smooth muscle relaxation or due to preservation of microvasculature or stimulation of angiogenesis. Future experiments with DPS-measurements of blood volume per cross-sectional area and measurements of vessel diameters might answer these questions.

The mechanism of increased pressure and overactivity reducing saturation that in turn reduces bladder function by impeding aerobic muscle action and by excitation of bladder nerves poses a self enhancing loop. Direct actions on the saturation part in this loop like with the PDE5 inhibitor sildenafil citrate used here may disrupt this loop and thereby might prevent the loss of bladder function that otherwise occurs.

## Conclusions

In a normal bladder BOS is high in the filling phase and drops slightly during voiding. In an obstructed bladder the loss of bladder function is accompanied by a significant decrease in BOS during voiding and filling. Treatment of obstructed bladders with SC maintains normal BOS during filling and voiding resulting in high bladder compliance and less DO. This supports the hypothesis that maintaining the microcirculation of the bladder wall results in better bladder performance without significant loss of bladder function. Measurement of BOS and interventions focussing on tissue microcirculation may have a place in the evaluation/treatment of various bladder dysfunctions.

## Abbreviations

BOO: Bladder outlet obstruction; BOS: Blood oxygen saturation; DPS: Differential path-length spectroscopy; NOC: Number of overactive contractions; PDE5: Phosphodiesterase 5; SC: Sildenafil citrate.

## Competing interests

The authors declare that they have no competing interests.

## Authors’ contributions

JRS participated in de conception and design of the study, and performed acquisition and analysis of the data, and performed drafting of the manuscript. AA performed acquisition and analysis of the data, and participated in critical revision of the manuscript. KPW performed acquisition of the data and participated in critical revision of the manuscript. DJK participated in de conception and design of the study, and performed acquisition and analysis of the data. All authors read and approved the final manuscript.

## Pre-publication history

The pre-publication history for this paper can be accessed here:

http://www.biomedcentral.com/1471-2490/14/44/prepub
